# The Link between Obstructive Sleep Apnea Syndrome and Cephalometric Assessment of Upper Airways and Hyoid Bone Position

**DOI:** 10.3390/medicina58091213

**Published:** 2022-09-03

**Authors:** Olja Tanellari, Celjana Toti, Edlira Baruti Papa, Sara Ghanim, Carmen Savin, Cristian Romanec, Carina Balcoș, Irina Nicoleta Zetu

**Affiliations:** 1 Department of Orthodontics, Faculty of Dental Medicine, “Grigore T. Popa” University of Medicine and Pharmacy, 700115 Iasi, Romania; oli_koca@yahoo.com (O.T.); cromanec@gmail.com (C.R.); nicoletazetu@gmail.com (I.N.Z.); 2 Department of Orthodontics, Faculty of Dental Medicine, University of Medicine, 1005 Tirana, Albania; celialmiro@hotmail.com; 3 Department of Dentistry And Maxillofacial Surgery, Salus Hospital, 1000 Tirana, Albania; andiaaura@gmail.com; 4 Operator for Healthcare Services (OSHKSH), 1000 Tirana, Albania; ghanimsara@yahoo.com

**Keywords:** hyoid bone position, lateral cephalogram, OSAS, upper airways

## Abstract

*Background and Objectives*: To determine whether there are changes in the cephalometric characteristics of the upper airways and hyoid bone, in patients diagnosed with obstructive sleep apnea syndrome (OSAS) compared to a healthy control group. *Material and Methods*: This retrospective case–control study included 24 patients diagnosed with OSAS (apnea–hypopnea index (AHI) > 5 obtained after polysomnography) and 24 healthy subjects as a control group that completed the STOP-Bang questionnaire to determine whether they had OSAS. Lateral cephalometric examinations were recommended for all these patients. The software used for the cephalogram interpretation was CS 3D Imaging and CS Airway imaging from Carestream Dental. *Results*: The subjects with OSAS had a smaller superior posterior airway space (SPAS), with an average of 10.32 mm compared to a 12.20 mm mean in the control group (*p* = 0.03). Patients with OSAS, had a lowered middle airway space (MAS) with a mean of 7.96 mm in the OSAS group and a 10.96 mm mean in the control group (*p* = 0.00). All the measurements made for the hyoid bone, such as—H-MnP, H-C3, and H-B—showed increased values (means of 26.31 mm, 39.08 mm, 60.05 mm, respectively), for the OSAS group (*p* = 0.00). *Conclusions*: Patients suffering from OSAS had reduced dimensions of the SPAS and MAS values. The hyoid bone had a more inferior position in the study group (with increased values for H-MnP, H-C3, and H-B) compared to the control group.

## 1. Introduction

The American Academy of Sleep Medicine (AASM) defines obstructive sleep apnea syndrome (OSAS) as a sleep-related breathing disorder characterized by a reduction (hypopnea) or cessation (apnea) in airflow despite an ongoing effort to breathe, caused by the collapse and obstruction of the upper airway. These respiration pauses, lasting at least 10 s, can also be accompanied by hypoxia and hypercapnia, which results in arousals from sleep to restore regular breathing. The apnea–hypopnea index (AHI) is used to determine the severity of sleep apnea based on the number of apneas and hypopneas experienced during one hour of sleep. AHI values exceeding five are deemed abnormal [1].

OSAS is a multifactorial illness associated with hyper-somnolence, cognitive impairment, metabolic dysfunction, and cardiovascular disease that poses a significant public health burden [2]. According to studies, this condition’s negative social and health-related consequences are correlated with worldwide financial distress [3]. Despite these negative effects, OSAS is still underdiagnosed [4,5].

OSAS’s pathophysiology is complex and multifactorial. It is caused by the combination of multiple factors, including age, sex, and overweight, but an abnormal dentofacial pattern (a dental-maxillary anomaly) is also considered to be a factor of significant influence.

The hyoid bone is crucial in keeping the airways free. Upper airway structures collapse in the supine position, and compensatory reflexes act on pharyngeal dilators holding the hyoid bone forward, aiding in preventing pharyngeal narrowing throughout the lifetime [6]. Some of the tongue muscle insertions are at the hyoid bone, therefore the position of this bone may be an important factor to be taken into consideration in obstructive sleep apnea syndrome. When this bone position is low, the tongue moves posteriorly, reducing airway volume. When compared to healthy patients, OSAS patients have a lower hyoid bone position in relation to other skeletal structures [7].

The gold standard for an accurate diagnosis of obstructive sleep apnea is Polysomnography (PSG). For a routine PSG, a thorough monitoring system is required to record sleep stages, limb movements, airflow, respiratory effort, heart rate and rhythm, oxygen saturation, and body posture [8]. Polysomnography cannot reveal the locations of airway obstructions, therefore computer tomography, magnetic resonance imaging, lateral cephalogram, and naso-laryngoscopy with an optic fiber can be used as diagnostic tools to detect airway obstructions. Lateral cephalogram and naso-laryngoscopy with optic fiber are diagnostic tools that aid in establishing the accurate location of the obstruction, which is a key element in treatment planning and prognosis in patients with OSAS [9,10].

OSAS-specific symptoms, such as mandibular retrognathia, mandibular micrognathia, increased values of the ANB angle(angle between lines NA and NB), lower position of the hyoid bone, and decrease in upper and lower pharynx space are cephalometric measures that have shown a greater association with the diagnosis of OSAS. Despite the above, the craniofacial features associated with OSAS vary across populations and ethnic groups [11,12].

Because obstructive sleep apnea is currently difficult to diagnose, sleep questionnaires, such as the Epworth Sleepiness Scale (ESS) [13] and the STOP-Bang questionnaire [14] have been very helpful tools for assessing potential sleep-related disorders. The scores on the ESS provide an evaluation of the subject’s general level of daytime sleepiness, ranging from low to very high levels in eight conceivable conditions. Subjects are asked to rate, on a scale from 0 to 3, how likely they are to nod off or fall asleep in each of the eight scenarios, depending on their typical lifestyle in the past few months. There is a contrast between falling asleep and feeling exhausted. If a person has not recently experienced some of the circumstances, he is nonetheless asked to estimate how each may affect him. The ESS attempts to overcome the reality that people have diverse daily routines, some of which facilitate daytime sleep and others that impede it [15].

The STOP-Bang questionnaire was created in response to the need for a simple, user-friendly obstructive sleep apnea screening tool in preoperative clinics. The questionnaire consists of four questions regarding snoring, fatigue, witnessed apnea, and hypertension. This questionnaire includes the four items from the STOP questionnaire plus four demographic questions concerning snoring, tiredness, observed apnea, high blood pressure, body mass index, age, neck circumference, and male gender [14]. The meta-analysis conducted by Chiu et al. [15] and by Nagappa et al. [16] suggests that the STOP-Bang questionnaire is an excellent screening instrument for obstructive sleep apnea.

Bayat et al. [17], Gosh et al. [18] and Neelapu et al. [19] have provided compelling evidence of an inferiorly positioned hyoid bone in OSAS patients. Consequently, the aim of this study is to determine whether there are changes in the cephalometric characteristics of upper airways and hyoid bone position in patients with OSAS, compared to a healthy control group.

## 2. Materials and Methods

### 2.1. Patients’ Selection

This case–control study was carried out on data collected from 24 patients diagnosed with OSAS (as validated by polysomnography investigation), and 24 subjects who did not suffer from this disease. The inclusion criteria were as follows: patients aged between 18 and 65 years; patients diagnosed with mild, moderate, or severe OSAS; patients that have provided informed consent. The exclusion criteria were as follows: patients aged under 18 or over 65 years old; patients who refused to sign the informed consent; patients with chronic obstructive pulmonary disease (COPD) or neurological or mental disorders that have been affected by upper respiratory tract infection; patients with a history of orthognathic surgery or any other type of respiratory surgery; patients with genetic syndromes and congenital malformations (lip–palate cleft, Pierre Robin syndrome and all other diseases with implications in the craniofacial skeletal structures); patients diagnosed with mild to moderate sleep apnea syndrome who have had a history of orthodontic treatment.

### 2.2. Study Design

In July 2022, data were obtained for this study from the observation charts of the patients who previously presented in two hospitals in Tirana, Albania for a variety of obstructive sleep apnea symptoms. Following a brief description of the study’s goal and methods, written informed consent was obtained from the patient. 

The patients in the OSAS group were selected from among the patients of the Maxillo-Facial Surgery department for orthodontic surgical treatment, they have the diagnosis of OSAS established based on AHI score > 5 obtained after polysomnography and on the detected changes by lateral cephalometry examinations. Based on the AHI, the severity of OSAS is classified as follows: none/minor: AHI < 5 per hour, mild: AHI ≥ 5 but < 15 per hour, moderate: AHI ≥ 15 but < 30 per hour< severe: AHI ≥ 30 per hour). Standardized lateral cephalogram evaluation was completed by the same technician. During the examination, the head was held in a neutral position and the teeth were in maximum intercuspation during the expiratory phase of breathing. All patients had normal body mass index (BMI).

The control group was selected from the patients with dental-maxillary anomalies who presented themselves at the Maxillo-Facial Surgery department for orthognathic surgery treatment. These patients completed the STOP-Bang questionnaire [16] to determine whether they had specific problems with obstructive sleep apnea.

Lateral cephalometric examinations were recommended for all these patients, based on the criteria listed in Table 1 and Table 2. The software used for the cephalogram interpretation was from Carestream Dental (Carestream Dental LLC, Atlanta, GA, USA), namely CS 3D Imaging and CS Airway imaging [20].

**Figure 1:**
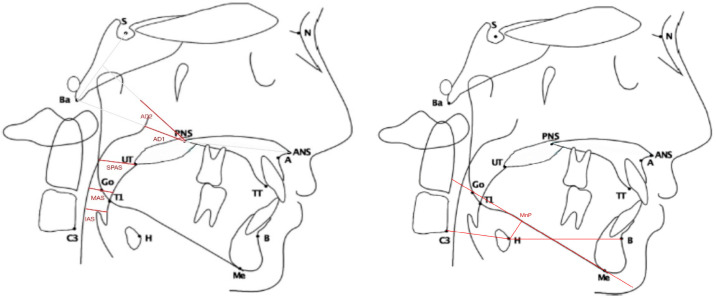
Representation of the cephalometric landmarks and references lines used for the airway space and hyoid bone measurements (sketches courtesy of Dr. Sara Ghanim) (S—point Sella; N—point Nasion; A—point A; ANS—anterior nasal spine; UT—tip of the uvula; T1—tongue base; TT—tip of the tongue).

Graphical explanations of points used in the measurements performed on lateral cephalogram in our study samples are presented in Figure 1.

### 2.3. Statistical Analysis

All the collected data were introduced into a Microsoft Excel program, from where they were then exported to SPSS (IBM SPSS Statistics, NY, USA) 25.0, a program in which all statistical analysis was performed. The statistical analysis used was descriptive and consisted of determining means and standard deviations for each group and each item analyzed. The Student’s independent t-test was used to compare the mean of cephalometric measurements between the two groups. A value of *p* < 0.05 was considered statistically significant.

## 3. Results

The study sample comprised 29 males (60.4%) and 19 females (39.6%). The mean age in the obstructive sleep apnea syndrome (OSAS) group was 41.88 (10.94) (95%CI, 37.26–46.49) and the mean age in the control group was 29.33 (11.87) (95%CI, 24.32–34.34) (Table 3).

Comparing the patients from the OSAS group with the ones from the control group, for the airway dimension evaluations, we obtained the following data: the PNS-AD2 distance had a mean of 21.84 in the OSAS group and a 21.28 mean in the control group. The difference between the means was 0.56 in favor of the OSAS group. The Sig. (2-tailed) showed no significant statistical correlation, with a value of 0.67.

The difference between the means was 2.22 in favor of the control group for the distance PNS-AD1 (34.93 in the OSAS group and 37.15 in the control group). The Sig. (2-tailed) showed no significant statistical relationship, with a value of 0.23.

The superior airway distance (SPAS) had a mean of 10.32 in the OSAS group and a 12.20 mean in the control group. The difference between the means was 2.22 in favor of the control group. The Sig. (2-tailed) showed a significant statistical relationship, with a value of 0.03.

Concerning the medium airway distance (MAS) it was observed that there was a difference between the means of 3, in favor of the control group (mean of 7.96 in the OSAS group versus a 10.96 mean in the control group). The Sig. (2-tailed) showed a significant statistical relationship, with a value of 0.00.

With respect to inferior airway distance (IAS), we observed a mean value of 10.97 for the OSAS group and a mean value of 11.17 for the control group. Therefore, the difference between the means was again in the favor of the control group with 0.2. The Sig. (2-tailed) showed no significant statistical relationship, with a value of 0.83 (Table 4).

Comparing the OSAS group and the control group, for the hyoid bone evaluations, we came to the following results. The distance between the hyoid bone and the B point, expressed as H-B, had a mean of 60.05 in the OSAS group and a mean of 50.96 in the control group. The difference between them was 9.09 in favor of the OSAS group. The Sig. (2-tailed) showed a significant statistical relationship, with a value of 0.00.

The distance between the hyoid bone and the mandibular plane, expressed as H-MnP, has a mean of 26.31 in the OSAS group and a mean of 16.17 in the control group. The difference between them was again in the favor of the values of the OSAS group, with 10.14. The Sig. (2-tailed) showed a significant statistical relationship, with a value of 0.00.

The distance between the hyoid bone and the third cervical vertebra point, expressed as H-C3, had a mean of 39.08 in the OSAS group and a mean of 34.97 in the control group. The difference between them was also in favor of the OSAS group (4.11). The Sig. (2-tailed) showed a significant statistical relationship, with a value of 0.00 (Table 5).

## 4. Discussion

The study carried out on a group of patients from Albania highlights the possibility of using lateral cephalometric examinations in the dental office as a screening tool for patients with OSAS, given the fact that polysomnography is a time-consuming, expensive technique and does not provide information on the area of obstruction [21].

Adult patients with OSAS present different craniofacial modifications, the maxilla, and the mandible is in a posterior position in comparison with the cranial base (retro-position), and the hyoid bone is in an inferior position in relation to the mandible or maxilla, with an increased facial height [22]. One of the highly potential diagnostic parameters of OSAS and its severity is the superior/inferior position of the hyoid bone. The inferior placement of the hyoid bone increases the likelihood of pharyngeal collapse [19].

There are several theories as to why the hyoid bone is positioned inferiorly in OSAS patients. This phenomenon, it has been claimed, is already present in a reduced capacity at the onset of OSAS [23]. This upper airway anomaly which can be highlighted on the lateral cephalogram is exacerbated by aging, weight gain, or external influences. This hypothesis is supported by the observed association between age or weight and hyoid bone position [24,25,26,27].

The most obvious change of craniofacial values found in this study was the inferior position of the hyoid bone in OSAS patients when compared with the control group (the H-MnP, H-C3 and H-B distances were increased in the OSAS group). Our results are in the line with the data from other studies which concluded that there is a direct correlation between OSAS and the hyoid bone position [28,29,30,31].

Overweight patients with OSAS present breathing problems because of the changes in the anatomy of the upper airway (fat deposition in the pharynx makes the upper airway narrower), changes in the function of the upper airway (mechanisms that control airway patency), an imbalance between respiratory drive and workload, and a decrease in functional residual capacity [32,33,34].

Weight gain cannot entirely be explained by inferior position of the hyoid bone, as the hyoid bone was also observed to be positioned more inferiorly in the less obese or non-obesity OSAS patients when compared to non-OSAS subjects. It has been postulated that the inferior position of the hyoid bone is a consequence of OSAS and is typically observed in patients with severe OSAS and a lengthy disease history [23].

The success of surgical advancement of the hyoid bone in the treatment of OSAS [35] confirms the hypothesized strong association between a more inferiorly positioned hyoid bone and greater OSAS severity. The tongue muscles are inserted into the hyoid bone and serve a crucial function in maintaining upper airway patency [36]. Indications of a shift in the location of the tongue and its musculature include an inferiorly located hyoid bone (caused, for example, by macroglossia, extensive fat deposits, or relaxation of the lingual muscle). Consequently, the hyoid bone has a more inferior position compared to the control group, based on the characteristics measured from the hyoid bone, according to other research (including the ones cited above) such as Bayat et al. [17] and Neelapu et al. [19]. In our investigation, all measurements of the hyoid bone, including H-MnP, H-C3, and H-B, yielded identical results, indicating an increase in the distance in the OSAS group.

There is substantial controversy as to whether the anatomy of the upper airway differs between the sexes. The results of cephalometric examinations of the pharyngeal region are unclear. Some research has found that the female pharynx is smaller than the male pharynx, however, this conclusion has not been supported by other studies [37,38,39,40].

The values obtained in the study conducted by us for the OSAS group were bigger in comparison with the values obtained in the study conducted by Bayat et al. [17] for SPAS (mean SPAS value of 11.43 vs. 10.32) and smaller for MAS (mean MAS value 7.96 vs. 8.79).

In comparison with the control group, the OSAS patients enrolled in our study had statistically significant reductions in their superior and middle airway space. There were no statistically significant variations between the data of the remaining airway space.

Despite the evolution of medicine, continuous positive airway therapy continues to be the gold standard in the treatment of patients with OSAS. Patients who do not tolerate continuous positive airway pressure (CPAP) may require a multidisciplinary approach to the OSAS which means a multidisciplinary team approach (sleep medicine specialist, pneumologist, ear–nose–throat specialist, orthodontist, and maxillo-facial surgeon) to determine the most accurate and appropriate therapeutic management. With this individualized approach, a correct patient selection can help identify and develop a treatment plan that may maximize the patient’s benefits.

The main limitations of this study are represented by the small number of participants in each group and by the age distribution between the two groups that were not homogeneous.

## 5. Conclusions

The subjects suffering from OSAS had a statistically significant lowered superior and middle airway space. The hyoid bone position was inferior in the patients with OSAS compared to the patients from the control group (H-MnP; H-C3; and H-B distances had an increased value for the OSAS group). The position of the hyoid bone on the lateral cephalogram can be used as a screening instrument in patients with symptoms of obstructive sleep apnea.

## Figures and Tables

**Table 1:** Criteria for airways space measurements.

Airway Evaluation	
Measurements	Explanations
AD1	Adenoid point 1 (adenoid tissue on the PNS-Ba line)
AD2	Adenoid point 2 (adenoid tissue on the midpoint between sella and basion to PNS line)
PNS-AD1	Lower nasopharyngeal airway space (width of airway along PNS-Ba line)
PNS-AD2	Upper nasopharyngeal airway space (width of airway along PNS-R line)
SPAS	Superior posterior airway space(width of airway behind soft palate along parallel line to palatal plane)
MAS	Middle airway space (with of airway along parallel line to palatal plane trough Sp)
IAS	Inferior airway space (width of airway along parallel line to palatal plane through epiglottis tip)

AD—adenoid point; PNS—posterior nasal spine; SPAS—superior posterior airway space; MAS—middle airway space; IAS—inferior airway space; Ba—point Basion; R—point located at the intersection of the posterior pharyngeal wall with the line between PNS and Hor point (intersection between the greater wing and the body of the sphenoid bone); Sp—tip of the soft palate.

**Table 2:** Criteria for hyoid bone measurements.

Hyoid Bone Evaluation	
Measurements	Explanation
H-B	Distance from H to point B
H-MnP	Distance from H to mandibular plane (Go-Me)
H-C3	Distance from H to C3

H—the most superior point on the anterior surface of the body outline of the hyoid bone; B—point B; MnP—mandibular plane (Go-Me); Go—point Gonion; Me—point Menton; C3—the most inferior and the most anterior point of the third cervical vertebrae.

**Table 3:** Mean age in the study groups.

				95 CI for Mean	
		Mean	SD	Lower Bound	Upper Bound
Age	OSAS Group	41.88	10.94	37.26	46.49
	Control Group	29.33	11.87	24.32	34.34

**Table 4:** The comparison of the airway evaluations between the OSAS group and control group.

Comparison of OSAS and Control Groups (Airway Space Evaluation)					
		Mean	Std. Deviation	Std. Error Mean	Sig. (2-Tailed)
PNS-AD2	Control group	21.28	5.57	1.14	0.670
	OSAS group	21.84	3.37	0.69	
PNS-AD1	Control group	37.15	3.41	0.70	0.230
	OSAS group	34.93	8.17	1.67	
SPAS	Control group	12.20	3.34	0.68	0.030
	OSAS group	10.32	2.44	0.50	
MAS	Control group	10.96	3.44	0.70	0.000
	OSAS group	7.96	1.59	0.33	
IAS	Control group	10.97	3.89	0.79	0.830
	OSAS group	11.17	2.31	0.47	

**Table 5:** The comparison of the hyoid evaluations of the OSAS group and control group.

Comparison of OSAS and Control Groups (Hyoid Bone Evaluation)					
		Mean	Std. Deviation	Std. Error Mean	Sig. (2-Tailed)
H-B	Control group	50.96	5.11	1.04	0.000
	OSAS group	60.05	4.36	0.89	
H-MnP	Control group	16.17	5.83	1.19	0.000
	OSAS group	26.31	5.34	1.09	
H-C3	Control group	34.97	3.43	0.70	0.000
	OSAS group	39.08	5.60	1.14	

## Data Availability

Not applicable.
